# Cell Division Control Protein 42 Interacts With Hepatitis E Virus Capsid Protein and Participates in Hepatitis E Virus Infection

**DOI:** 10.3389/fmicb.2021.775083

**Published:** 2021-11-01

**Authors:** Mengnan Fan, Yuhang Luo, Beibei Zhang, Jiaxi Wang, Tianxiang Chen, Baoyuan Liu, Yani Sun, Yuchen Nan, Julian A. Hiscox, Qin Zhao, En-Min Zhou

**Affiliations:** ^1^Department of Preventive Veterinary Medicine, College of Veterinary Medicine, Northwest A&F University, Xianyang, China; ^2^Institute of Infection, Veterinary and Ecological Sciences, University of Liverpool, Liverpool, United Kingdom

**Keywords:** hepatitis E virus (HEV), avian HEV, CDC42, Rho GTPases, virus-host interaction

## Abstract

Hepatitis E Virus (HEV) causes viral hepatitis in humans worldwide, while a subset of HEV species, avian HEV, causes hepatitis-splenomegaly syndrome in chickens. To date, there are few reports on the host proteins interacting with HEV and being involved in viral infection. Previous pull-down assay combining mass spectrometry indicated that cell division control protein 42 (CDC42), a member belonging to the Rho GTPase family, was pulled down by avian HEV capsid protein. We confirmed the direct interaction between CDC42 and avian and mammalian HEV capsid proteins. The interaction can increase the amount of active guanosine triphosphate binding CDC42 state (GTP-CDC42). Subsequently, we determined that the expression and activity of CDC42 were positively correlated with HEV infection in the host cells. Using the different inhibitors of CDC42 downstream signaling pathways, we found that CDC42-MRCK (a CDC42-binding kinase)-non-myosin IIA (NMIIA) pathway is involved in naked avian and mammalian HEV infection, CDC42-associated p21-activated kinase 1 (PAK1)-NMIIA/Cofilin pathway is involved in quasi-enveloped mammalian HEV infection and CDC42-neural Wiskott-Aldrich syndrome protein-actin-polymerizing protein Arp2/3 pathway (CDC42-(N-)WASP-Arp2/3) pathway participates in naked and quasi-enveloped mammalian HEV infection. Collectively, these results demonstrated for the first time that HEV capsid protein can directly bind to CDC42, and non- and quasi-enveloped HEV use different CDC42 downstream signaling pathways to participate in viral infection. The study provided some new insights to understand the life cycle of HEV in host cells and a new target of drug design for combating HEV infection.

## Introduction

Hepatitis E virus (HEV), a member of the family *Hepeviridae*, is a potential public health issue and includes viruses belonging to two genera, *Orthohepevirus* and *Piscihepevirus* (Smith et al., 2014). HEV is a positive-sense RNA virus with a complete genome length of approximately 7.2 kb. The *Orthohepevirus* genus encompasses the greatest number of HEV isolates, which have been assigned to four species designated A, B, C, and D. *Orthohepevirus* A can infect a wide range of mammalian species (Lee et al., 2016; Kenney and Meng, 2019) and exists in nature as two different forms of viral particles: non-enveloped (naked) and quasi-enveloped virions. Naked virions are present in bile and feces, while virions coated with host plasma membranes are found in blood and supernatants of infected cell cultures (Okamoto, 2013). Avian HEV, a member of the species *Orthohepevirus* B, is the causative agent of the hepatitis-splenomegaly syndrome and big liver and spleen disease in chickens, leading to high mortality rates and decreased layer and breeding hen egg-production rates (Kenney and Meng, 2019).

The complete HEV genome consists of three open reading frames (ORF): ORF1, ORF2, and ORF3. The ORF2-encoded viral capsid protein generally includes 660 amino acids (aa), although in avian HEV it is only 606 aa in length (Zhao et al., 2015; Fu et al., 2019), it plays a crucial role during viral infection by interacting with host factors (Kamar et al., 2009; Shukla et al., 2011; Kapur et al., 2012; Ahmed et al., 2016; Yin et al., 2016, 2017; Li et al., 2019; Sayed et al., 2020). Due to the lack of a highly efficient *in vitro* cell culture system, two truncated HEV capsid proteins spanning 112–606 aa (Kamar et al., 2009) and 368–606 aa (designated p239) were universally used to screen the host proteins interacting with HEV based on both truncated capsid proteins mimic non-enveloped HEV particles (Yamashita et al., 2009). To date, several host factors interacting with HEV capsid protein were identified, including Grp78, α-tubulin, heat shock protein 90, cytochrome P4502C8, heparin surface proteoglycans, ATP synthase subunit β, and integrin α3 (Kamar et al., 2009; Zheng et al., 2010; Shen et al., 2011; Yu et al., 2011; Ahmed et al., 2016; Shiota et al., 2019). These host proteins were involved in the different stages of HEV infection in the cells. Nevertheless, the life cycle of HEV in host cells is still not clarified clearly.

Previously, a truncated avian HEV capsid protein spanning aa 313–549 (designated ap237) with sequence homology to HEV p239 served as a bait protein to screen for host interaction partners of avian HEV capsid protein (Li et al., 2019). Among screened host factors, organic anion transporting polypeptide 1A2 (OATP1A2) was shown to enhance avian HEV infection of host cells, prompting the creation of a cell line (designated LMH^OATP1A2^) that expressed OTAP1A2 (Li et al., 2019). Meanwhile, another host factor, a small Rho family GTPase, known as cell control protein 42 (CDC42), was also pulled down by ap237 (Li et al., 2019). CDC42 is known to moderate cellular actin dynamics and is the primary upstream triggering molecule of CDC42 signaling pathways (Doherty and McMahon, 2009; Swaine and Dittmar, 2015; Antkowiak et al., 2016). Notably, CDC42 signaling pathways are associated with infections of many viruses in host cells, including the Lassa virus (Oppliger et al., 2016; Fedeli et al., 2017), porcine reproductive and respiratory syndrome virus (Wei et al., 2020), enterovirus (Dun et al., 2020), transmissible gastroenteritis virus (Hu et al., 2016), porcine hemagglutinating encephalomyelitis virus (Lv et al., 2018), respiratory syncytial virus (Krzyzaniak et al., 2013), dengue virus (Chou et al., 2016), human immunodeficiency virus (Krautkramer et al., 2004; Rojek et al., 2008; Nikolic et al., 2011; Dutartre et al., 2016), Japanese encephalitis virus (Khasa et al., 2019), Ebola virus (Lu et al., 2013), Epstein-Barr virus (Tugizov et al., 2013), and adeno-associated virus (Weinberg et al., 2014). We investigated the relationship between CDC42 and HEV infection in host cells and found that CDC42 can directly interact with mammalian and avian HEV capsid proteins. Moreover, the expression level and activity of CDC42 were shown to be positively correlated with HEV infection. Subsequent identification of specific CDC42 signaling pathways participating in naked and quasi-enveloped HEV infection indicated that different forms of HEV virions exploited different CDC42 signaling pathways. Specifically, the CDC42-MRCK-NMIIA pathway is involved in non-enveloped HEV virions, the CDC42-PAK1-NMIIA/Cofilin pathway is involved in quasi-enveloped HEV virion, and the CDC42-(N-)WASP-Arp2/3 pathway is involved in both non- and quasi-enveloped HEV virions. Our findings provide new insights to understand the process of HEV infection in host cells while also identifying targets to guide the development of future novel therapies to prevent or combat HEV infection.

## Materials and Methods

### Cells and Viruses

LMH ^OATP1A2^ (LMH stably expressing OATP1A2) cell line was separately constructed and developed as previously described (Li et al., 2019). HepG2/C3A and HEK 293T cell lines were purchased from American Type Culture Collection. HEK 293T and LMH^OATP1A2^ cells were propagated in Dulbecco’s modified Eagle’s medium (DMEM; Gibco, Grand Island, NY, United States) supplemented with 10% fetal bovine serum (FBS; Biological Industries, Kibbutz Beit Haemek, Israel), 100 U/mL penicillin (Life Technologies Corp., Grand Island, NY, United States), and 100 μg/mL streptomycin (Life Technologies Corp.). HepG2/C3A cells were cultured in Minimum Essential Medium (MEM; Gibco) supplemented with 10% FBS. All cells were cultured at 37°C with 5% CO_2_.

HEV-3 strain Kernow C1/p6 (GenBank accession no. JQ679013) was generously provided by Suzanne U. Emmerson and propagated in HepG2/C3A cells (Shukla et al., 2012). Avian HEV stock was prepared from fecal suspensions obtained from SPF chickens intravenously inoculated with a clinical bile sample containing avian HEV from a 35-week-old breeder chicken in China (CaHEV, GenBank accession no. GU954430).

### Antibodies and Reagents

All reagents were purchased commercially unless otherwise indicated, as follows: anti-HA tag mouse monoclonal antibody (mAb), anti-His tag mAb, anti-tubulin mAb, and horseradish peroxidase (HRP)-conjugated goat anti-mouse IgG antibody (TransGen Biotech, Beijing, China); anti-HEV ORF2 protein mAb (Clone no. 1E6) (Sigma-Aldrich, St. Louis, MO, United States); rabbit anti-MRCKβ polyclonal antibody (pAb) (Invitrogen, Carlsbad, CA, United States); rabbit anti-HA tag pAb, anti-Flag tag pAb, anti-RAC1 pAb, anti-CDC42 pAb, anti-RAC1 pAb, anti-RhoA pAb, anti-PAK1 pAb, anti-Arp3 pAb, anti-MYLK pAb, anti-MYH9 pAb, and anti-MLC 2V pAb (Protein Tech, Rosemont, IL, United States); HRP-conjugated goat anti-rabbit or anti-chicken IgG antibody and fluorescein isothiocyanate (FITC)-conjugated goat anti-rabbit IgG antibody (Jackson ImmunoResearch Laboratories, West Grove, PA, United States); ML141 and wiskostatin (Calbiochem, San Diego, CA, United States); ML7 (Abcam Cambridge, MA, United States); NSC23766 (Sigma Aldrich); IPA-3 and Fasudil (Selleck Chemicals, Houston, TX, United States); blebbistatin (TOCRIS Bioscience, Houston, TX, United States). The mAbs against HEV ORF2 protein (Clone nos. 1B5, 1H5, and 3E8) were produced as previously described (Dong et al., 2011; Liu et al., 2014; Li et al., 2018).

### Plasmids

CDC42 gene (GenBank accession no. XM_0152968262) was amplified from liver mRNA from an SPF chicken. CDC42 genes were cloned into the pET-28a (+) (Novagen, Hornsby Westfield, Australia) using primers aCDC42-F/R to generate a fusion protein containing a His-tag. Plasmid pCAGEN-HA-CDC42 was constructed using primers aCDC42-F/R with the pCAGEN vector (Addgene, Watertown, MA, United States) to encode a fusion protein with a HA tag. Plasmid pLVX-ZsGreen1-CDC42 was constructed using primers lenti-aCDC42-F/pLVX-aCDC42-R with the pLVX-IRES-ZsGreen1 vector (TaKaRa) encoding a fusion protein with a Flag tag. Recombinant plasmids pCAGEN-ap237, pET-21b(+)-ap237, pET-21b(+)-ker239, pET-21b(+)-sar239, pET-21b(+)-rp239, and pET-21b(+)-sp239 were constructed as previously reported (Chen et al., 2018; Li et al., 2019). All positive plasmids were verified by sequencing conducted by Sango Biotech Co., Ltd. (Shanghai, China). Primers used in the study are listed in Table 1.

**TABLE 1:** Primers used in gene expression studies.

Primers name	Sequence (5′–3′)
aCDC42-F	GAATTCATGCAGACGATTAA
aCDC42-R	CTCGAGTAGCAGCACACACC
lenti-aCDC42-F	GAATTCGCCGCCACCATGCAGACGATTAAGTGTGT
pLVX-aCDC42-R	CTCGAGTCACTTATCGTCGTCATCCTTGTAATCTA GCAGCACACACCTGCGA
CDC42-qF	ATTAAGTGYGTWGTTGTGGGY
CDC42-qR	TCATMACTGTKACWGCATAGTT
GAPDH-gallus-qF	AAACTCATTGTCATACCAGG
GAPDH-gallus-qR	ATACACAGAGGACCAGGTTG
CaHEV-Taqman-F	TATGTGCTGCGGGGTGTCAA
CaHEV-Taqman-R	CATCTGGTACCGTGCGAGTA
Probe-ORF3-CaHEV	FAM-CTCCCAAACGCTCCCAGCCGGA-BHQ
EF1	ATGTTGGTGGGGTGCTGGTCGAGATTG
ER1	GGGTTGATTGGTCCGATATGATGCCAG
EF2	TTGTTGGACATACCCCCGGCCCACA
ER2	TAATCACCGCAAGACGGCTAGTGG
HEV-ORF1-qF	GTTGAGCAGAACCCGAAGAG
HEV-ORF1-qR	CGGGCTCAGTCAAGTAAAGC
HEV-ORF3-qF	GGTGGTTTCTGGGGTGAC
HEV-ORF3-qR	AGGGGTTGGTTGGATGAA
Probe-ORF3-HEV	FAM-TGATTCTCAGCCCTTCGC-BHQ
GAPDH-qF	ACAAGGCTGGGGCTCATTTG
GAPDH-qR	AGGGGCCATCCACAGTCTTC

### Protein Expression in Bacteria

Procedures for inducing expression of ap237, sar239, ker239, rp239, and sp239 without any tags and His-tagged CDC42 (CDC42-His) were based on the modified method reported by Chen et al. (2018). Briefly, bacterial expression of proteins was induced by adding 1 mM IPTG followed by incubation for 6 h at 37°C. Then, bacterial cells were harvested and lysed by sonication. Proteins were dissolved in 8 M urea, filtered through a 0.22 μM filter, and then refolded during dialysis against a series of buffers consisting of 6 M urea, 4 M urea, and 2 M urea 0.01 M phosphate buffer (PB) at pH 7.5. Refolded proteins were purified using a Superdex 200 Increase 10/300 GL Column (GE Healthcare, New Jersey, United States) connected to an AKTA Purifier System (GE Healthcare). The recombinant VP2 protein of infectious bursal disease virus (IBDV VP2) served as the negative control and was expressed and purified according to the abovementioned procedures.

### Co-immunoprecipitation and Pull-Down Assays

Plasmids pCAGEN-ap237 and pCAGEN-HA-CDC42 were co-transfected into cells, and then cells were collected at 48 h post-transfection (hpt). The cell pellet was lysed in NP-40 cell lysis buffer (Beyotime, Beijing, China) containing 1 × protease and phosphatase inhibitor cocktail (Beyotime) for 30 min on ice, and then the lysate was centrifuged at 13,000 × *g* at 4°C. Out of 600 μL of supernatant, 40 μL was set as the input group (namely, 6.7% of total proteins), and the additional supernatant was trisected and incubated with 1H5 mAb or anti-HA mAb and Dynabeads Protein G (Invitrogen) at 4°C for 6 h. To detect direct interactions among CDC42 and truncated capsid proteins, CDC42-His (10 μg) and each truncated capsid protein (ap237, ker239, sar239, sp239, or rp239, 10 μg/each) were co-incubated in PBS (pH 7.2) at 4°C overnight. Next, each mixture was divided into three equal parts based on volume, and the three portions were incubated at 4°C for 6 h with Dynabeads Protein G coated with either 3E8 mAb, anti-His mAb, or control Mouse IgG. Protein-bound beads were then collected and washed three times with PBS (pH 7.5) containing 0.02% Tween-20. Finally, all samples were analyzed by Western blotting.

### ELISA

To verify direct interactions among CDC42 and HEV capsid proteins, ELISAs were performed. Briefly, 96-well ELISA plates (Nunc Immunoplates, Nunc, Roskilde, Denmark) were coated with ap237 at various concentrations (10, 1, 0.1, or 0.01 μg/well) or CDC42-His (8, 4, 2, or 1 μg/well) and incubated at 4°C overnight. After washing wells with PBS containing 0.5% w/v Tween-20 (PBST) and blocking wells with PBST containing 1% BSA, CDC42-His, or HEV capsid proteins (1 μg/well) were added to plate wells, respectively. And then, plates were incubated for 1 h at 37°C. After washing three times, anti-His mAb (1:5,000) or 3E8 mAb (1:1,000) were added, respectively. Then, plates were incubated and washed again, as mentioned above. Finally, HRP conjugated goat anti-mouse IgG secondary antibody (1:5,000) was added into the wells. After another three washes, tetramethylbenzidine was added, the plates were incubated for 15 min then the colorimetric reaction was stopped by adding 3 M H_2_SO_4_. IBDV-VP2 protein was set as the negative control of CDC42-His protein because the vector and the tag of the two proteins were the same, while BSA was set as the negative control of the truncated capsid proteins of HEV because these proteins did not generate with the His-tag fusion. The OD_45__0__n__m_ value was read using an automated ELISA plate reader (Bio-Rad, CA, United States). Meanwhile, anti-avian HEV IgG antibodies in sera from challenged SPF chickens were detected using ELISA according to the method of Zhao et al. (2013).

### Indirect Immunofluorescence Assay

For analyzing the co-localization of ap237 and CDC42, cells were fixed with 4% paraformaldehyde for 20 min at 37°C, permeabilized with 0.25% Triton X-100 for 15 min at 37°C, then blocked with PBS containing 1% BSA for 1 h after co-transfection for 48 h. Next, cells were separately incubated with rabbit anti-HA pAb and 3E8 mAb for 1 h at 37°C and followed by FITC-conjugated goat anti-rabbit IgG (green) and Alexa Fluor^®^ 555-conjugated goat anti-mouse IgG (red, Invitrogen) for an additional 1 h. Finally, cells were stained with Fluoroshield^TM^ with DAPI (Sigma-Aldrich) and observed with a Leica SP8 confocal system (Leica, Wetzlar, Germany). Co-localization of ap237 and CDC42 was confirmed after Pearson’s correlation (Rr) and Manders’ overlap coefficient (R) data analyses were conducted using Image J (National Institutes of Health, United States) and Image-pro Plus^®^ (Media Cybernetics, United States). Both coefficients indicated actual overlaps of fluorescence signals. For visualization of HEV-positive cells, 1E6 mAb was used as the primary antibody. All images were captured and processed using Leica Application Suite X (Version 1.0. Leica Microsystems).

### Cell Division Control Protein 42 Pull-Down Activation Assay

The activation assay *in vitro* was carried out according to the manufacturer’s instructions of the CDC42 Pull-down Activation Assay Kit (Cytoskeleton, CO., United States). Briefly, 300 μg of CDC42-His was incubated with 50 μg of either ap237 or ker239, and then a 1/100 volume of GTPγS was immediately added to each group. Next, sample mixtures were incubated at room temperature for 15 min with gentle rotation. The reaction was stopped, and the samples were immediately added to PAK-PBD beads (10 μg of beads per sample) to pull down GTP-CDC42. Each sample with the beads was incubated at 4°C for 1 h, and the beads were washed twice and immunoblotted with rabbit anti-CDC42 pAb.

Since CDC42 proteins are generally activated very rapidly from 30 s to 30 min (Petermann et al., 2009; Quetglas et al., 2012), four-time points within 30 min were chosen to detect GTP-CDC42 in host cells during either ap237 (or ker239) incubation or CaHEV (or HEV-3) inoculation. Briefly, the cells were seeded into T25 flasks for 24 h before either ap237 (or ker239) or CaHEV (or HEV-3) were added into each flask. After washing, cells were collected at 0, 10, 20, and 30 min for GTP-CDC42 analysis. Briefly, 800 μg of cell lysis supernatant of each group were incubated with 10 μg of PAK-PBD beads at 4°C for 1 h, and the beads were washed twice and analyzed by Western blotting.

### Real-Time RT-qPCR (qPCR)

As previously described, 16 liver tissues from CaHEV-positive chickens and three liver tissues from HEV-negative SPF chickens were collected (Liu et al., 2017). Total RNA was extracted using TRIzol Reagent (TaKaRa, Tokyo, Japan). CaHEV and HEV-3 virus copy numbers were quantified as reported previously (Jothikumar et al., 2006; Troxler et al., 2011) using QuantiTect^®^ Probe RT-PCR kit (QIAGEN, Duesseldorf, Germany) with an Applied Biosystem StepOnePlus^TM^ Real-Time PCR System (Applied Biosystems, CA, United States). cDNAs were prepared from total RNA isolated from each group using random primers and a cDNA kit (TaKaRa, Tokyo, Japan) to measure mRNA abundance to detect relative numbers of virus particles. All specific primers used in the assay are shown in Table 1.

### RNA Interference

All siRNAs targeting CDC42, RAC1, RhoA, Arp3, PAK1, MYLK, and MYH9, respectively, and the siRNA-negative control (siNCtrl) were designed and synthesized by GenePharma (Shanghai, China). LMH^OATP1A2^ cells were transfected with siRNAs using Lipofectamine^TM^ RNAiMAX (Invitrogen) for 24 h. Subsequently, the expression levels of the indicated protein in transfected cells were analyzed by qPCR and Western blotting, respectively. It is worth noting that 500 nM of ap237 were pre-incubated with LMH^OATP1A2^ cells for Western blotting before transfection with siCDC42, siRAC1, and siRhoA, respectively. The siRNAs mentioned above are listed in Table 2.

**TABLE 2:** The siRNA targeting CDC42 in this study.

Name	5′–3′ (sense)	5′–3′(antisense)
siNCtrl	UUCUCCGAACGUGUCACGUTT	ACGUGACACGUUCGGAGAATT
siCDC42-323	CCAUCGGAAUACGUACCAATT	UUGGUACGUAUUCCGAUGGTT
siCDC42-578	GGGACCCAAAUUGAUCUAATT	UUAGAUCAAUUUGGGUCCCTT
siCDC42-721	GCAGAAAGGCCUAAAGAAUTT	AUUCUUUAGGCCUUUCUGCTT
siRAC1	GCAGUGAAAUACCUAGAAUTT	AUUCUAGGUAUUUCACUGCTT
siRhoA	CCGGAAGUGAAGCAUUUCUTT	AGAAAUGCUUCACUUCCGGTT
siPAK1	GGAUGGCUCUGUCAAAUUATT	UAAUUUGACAGAGCCAUCCTT
siMYLK	GGGACGAUGAUGCCAAAUATT	UAUUUGGCAUCAUCGUCCCTT
siMYH9	GGCCAAGGAAGAAGAACUATT	UAGUUCUUCUUCCUUGGCCTT
siArp3	GGCGUCCAUUAUAUAAGAATT	UUCUUAUAUAAUGGACGCCTT

### Viral Infection

Viral infection assays were performed according to the method of Oppliger et al. (2016). Briefly, after cells were cultured for 24 h in 12-well plates, they were transfected with CDC42-overexpressing plasmid, siCDC42-721, siRhoA, siRAC1, siPAK1, siMYLK, siNMHC, and siArp3, respectively, for another 24 h or were treated with different concentrations of the mentioned inhibitors of Rho GTPases family for 4 h. Then, the cells were inoculated with CaHEV (3.5 × 10^6^ copies/wells) or HEV-3 (6 × 10^6^ copies/well). After inoculation, the cells were washed thrice with PBS and were collected at 7 dpi for CaHEV and 5 dpi for HEV-3. Additionally, the negative-strand ORF1 RNA of CaHEV was also detected according to the method of Billam et al. (2008) using primer pairs EF1/ER1 and EF2/ER2 (Table 2) to confirm viral replication in LMH^OATP1A2^ cells.

### Envelope Removal to Generate Non-enveloped Hepatitis E Virus

Non-enveloped HEV was obtained using methods reported previously by Nagashima et al. (2014) and Yin et al. (2016). First, each culture supernatant containing HEV virions was concentrated by ultracentrifugation at 100,000 g for 2 h at 4°C. Next, virions were treated with 0.1% w/v sodium deoxycholate and 0.1% w/v trypsin at 37°C for 4 h. Non- and quasi-enveloped virions were generated by equilibrium centrifugation in isopycnic gradient centrifugation at 160,000 g in an SW41i rotor for 16 h at 4°C (Yin et al., 2016). After gradients were fractionated, both virus density and load in each fraction were measured using refractometry and qPCR.

### Cell Viability Analysis

Cell viability was evaluated by the CCK-8 (Beyotime) assay described previously with modifications (Chen et al., 2017). LMH^OATP1A2^ cells (5 × 10^4^ cells/well) and other cell lines (1 × 10^4^ cells/well) were seeded into 96-well plates and incubated with different concentrations of specific inhibitors of members of the Rho GTPase family. Next, the CCK-8 reagent (10 μL/well) was added and incubated for 1 h at 37°C. Finally, the OD_45__0__n__m_ value was read and used to calculate cell viability. Data are expressed as the percentage of the optical density of treated cells relative to that of the untreated control cells (controls defined as having 100% viability).

### Statistical Analysis

Each experiment was independently repeated at least three times. Data were presented as the mean ± standard deviation (SD). Statistical significance was determined by Student’s *t*-test or Kolmogorov-Smirnov test when two groups were compared using GraphPad (Graph-Pad Software Inc., San Diego, CA, United States). Pearson’s correlation coefficient (Rr) and overlap coefficient (R) was finished using Image-Pro Plus 6.0 software to assess the relationship between CDC42 and ap237. Moreover, Nair’s test was used to analyze outliers or stragglers of the data in the present study, and the outliers were removed, and the straggler was indicated as a hashtag. Asterisks indicate statistical significance as follows: ns, not significant; ^∗^ and a, *P* < 0.05; ^∗∗^ and aa, *P* < 0.01; ^∗∗∗^ and aaa, *P* < 0.001.

## Results

### Cell Division Control Protein 42 Directly Interacts With Avian Hepatitis E Virus Capsid Protein

**FIGURE 1:**
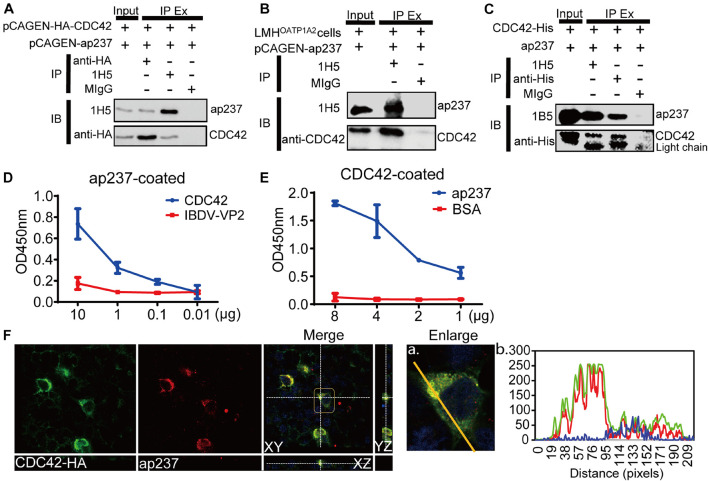
CDC42 interacts with the truncated capsid protein of avian HEV (designated ap237). **(A)** Co-IP of ap237 and exogenous HA-tagged CDC42 (HA-CDC42) in HEK 293T cells. HEK 293T cells were co-transfected with pCAGEN-ap237 and pCAGEN-HA-CDC42. The lysed cells were immunoprecipitated using anti-HA mAb, 1H5 mAb, and normal mouse IgG (MIgG), respectively, and then immunoblotted with either 1H5 mAb or anti-HA mAb. MIgG was set as a blank control. **(B)** Co-IP of ap237 and endogenous CDC42 in LMH^OATP1A2^ cells. 1H5 mAb was used to detect ap237 and Rabbit anti-CDC42 pAb to detect endogenous CDC42. **(C)** CDC42-His directly interacting with ap237 was determined by pull-down assay using anti-His and 1H5 mAb. The content of “the input” group was about 6.7% of the total proteins. **(D,E)** Confirmation of direct interaction between CDC42 and ap237 by indirect ELISA with ap237 as coated antigen **(D)** and recombinant CDC42-His protein as coated antigen **(E)**. **(F)** Co-localization of ap237 and CDC42 in LMH^OATP1A2^ cells. After co-transfection of pCAGEN-HA-CDC42 and pCAGEN-ap237 in the LMH^OATP1A2^ cells for 48 h, the cells were fixed and stained with anti-HA pAb and 3E8 mAb. The location of CDC42 (green) and ap237 (red) was analyzed by confocal microscopy. The nucleus is indicated by DAPI (blue) staining in the images. Outline regions are magnified (inset) **(A)**, and profiles of fluorescence intensity along the yellow line in corresponding images are shown in right panels analyzed using Image J software **(B)**.

The interaction between ap237 and CDC42 was confirmed by co-immunoprecipitation (Co-IP) assays and confocal microscopy-based observations. The results showed that CDC42-HA and ap237 proteins were specifically pulled down together without being pulled down by normal mouse IgG (MIgG) (Figure 1A). Subsequently, the interaction between endogenous CDC42 and ap237 was also detected in LMH^OATP1A2^ cells. The endogenous CDC42 in the LMH^OATP1A2^ cells was pulled down by ap237 (Figure 1B). Next, to examine whether CDC42 could directly interact with ap237, ap237, and CDC42 fused with a His-tag (CDC42-His) protein were expressed using the prokaryotic expression system. SDS-PAGE analysis showed that the two proteins were successfully expressed and purified (Supplementary Figure 1A). Then, using the purified two proteins for pull-down experiments and enzyme-linked immunosorbent assays (ELISAs), the results showed that ap237 bound to CDC42-His in solution (Figure 1C) as well as to solid phase-immobilized CDC42-His in a specific, dose-dependent manner (Figures 1D,E). Moreover, using confocal microscopy experiments to assess co-localization of ap237 and CDC42-HA in cells, the results revealed that ap237 (red) was present throughout the cytoplasm and co-localized with CDC42-HA (green) in HEK 293T cells (Supplementary Figure 1B, mean Rr: 0.895 ± 0.161; and R: 0.921 ± 0.252). The similar results were obtained in LMH^OATP1A2^ cells (Figure 1F, mean Rr: 0.871 ± 0.102; R: 0.905 ± 0.150). Collectively, the abovementioned results indicated that CDC42 directly interacted with avian HEV capsid protein.

### Levels of GTP-Cell Division Control Protein 42 Increase After Avian Hepatitis E Virus Infects Host Cells

**FIGURE 2:**
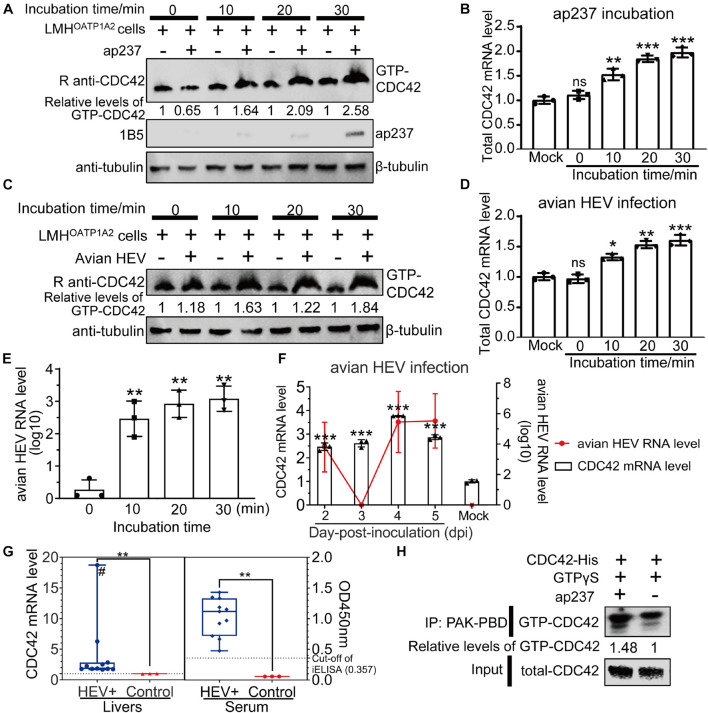
Expression levels of total GTP-CDC42 and CDC42 in ap237 treated and CaHEV infected cells. **(A,B)** Expression levels of GTP-CDC42 and mRNA of total CDC42 in the LMH^OATP1A2^ cells treated with ap237 for 0, 10, 20, and 30 min. **(A)** Lysates of each group were incubated with PAK-PBD beads to pull down GTP-CDC42, then the beads were washed twice and immunoblotted with rabbit anti-CDC42 pAb. β-tubulin was employed as an internal control. **(B)** The relative RNA levels of the total CDC42 of each group were determined by qPCR. **(C,D)** Expression levels of GTP-CDC42 and mRNA of total CDC42 in the LMH^OATP1A2^ cells inoculated with CaHEV for 0, 10, 20, and 30 min. **(E)** The amounts of avian HEV by qPCR in LMH^OATP1A2^ cells at 0, 10, 20, and 30 min-post-inoculation. **(F)** CDC42 mRNA levels in liver tissues from chickens inoculated with CaHEV at 2, 3, 4, and 5 dpi. **(G)** Relative mRNA levels of CDC42 in CaHEV-positive chicken livers (left panel) and anti-avian HEV IgG levels in sera corresponding to CaHEV-positive chicken livers (right panel). The symbol “#” means that the value is a straggler. **(H)** CDC42 binding more GTPs through interaction with ap237 *in vitro*. The mixtures of CDC42-His and ap237 or only CDC42-His were incubated with GTPγS at room temperature. After stopping the reaction, the samples were immediately added to 10 μg of PAK-PBD beads and incubated at 4°C for 1 h, and beads were washed twice and analyzed by Western blotting. CDC42 mRNA levels were normalized to avian GAPDH. ns, no significant; *P < 0.05; **P < 0.01; ***P < 0.001.

Expression levels of total CDC42 were analyzed after CaHEV infection *in vitro* and *in vivo*. It had been previously reported that CDC42 activated signaling pathways after its conversion from an inactive GDP-bound state (GDP-CDC42) to an active GTP-bound state (GTP-CDC42) (Quetglas et al., 2012). So, the relationship between GTP-CDC42 and avian HEV infection was also determined. The results showed that protein levels of GTP-CDC42 and mRNA levels of total CDC42 increased gradually after the LMH^OATP1A2^cells were treated with ap237 and by CaHEV inoculation for 10, 20, and 30 min (Figures 2A–D). Avian HEV RNA was detected in the LMH^OATP1A2^ cells, indicating that avian HEV successfully infected the cells (Figure 2E). *In vivo*, total CDC42 levels in livers from CaHEV-inoculated chickens also increased gradually at 2, 3, 4, and 5 day-post-inoculation (dpi) (Figure 2F), while total CDC42 levels in livers from infected chickens significantly exceeded levels observed in uninoculated specific-pathogen-free (SPF) chickens (Figure 2G). Furthermore, to analyze the relationship between GTP-CDC42 levels and interaction of CDC42 with ap237, CDC42 activation assays *in vitro* were performed. The results showed that more GTP-CDC42 was pulled down when ap237 was added into the mixture of CDC42 and GTPγS, suggesting that the interaction between CDC42 and avian HEV capsid protein promoted CDC42 binding to GTP (Figure 2H). Altogether, these results indicated that total CDC42 and GTP-CDC42 increased after host cells were infected with avian HEV.

### Cell Division Control Protein 42 Is Involved in Avian Hepatitis E Virus Infection in LMH^OATP1A2^ Cells

**FIGURE 3:**
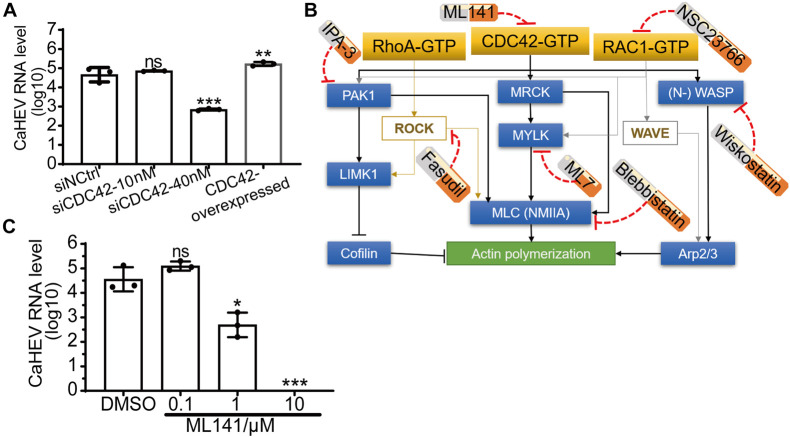
Positive correlation between expression level and activity of CDC42 and avian HEV infection in host cells. **(A)** Copies of CaHEV RNA in CDC42-overexpressed and -knocked down LMH^OATP1A2^ cells after inoculated with CaHEV at 7 dpi. **(B)** Schematic diagrams of different small molecular inhibitors for Rho family GTPases. ML141 is a selective, reversible non-competitive inhibitor of CDC42, preventing GTP binding to the active site of CDC42 without targeting any CDC42-regulating molecules. Fasudil is a non-specific RhoA-associated kinase (ROCK) inhibitor. NSC23766 is an inhibitor of RAC1 activation. IPA-3 is a downstream CDC42 signaling inhibitor, selectively inhibiting group 1 PAKs by targeting the auto-regulatory mechanism. ML7 potently inhibits myosin light chain kinase (MLCK). Blebbistatin is a selective non-muscle myosin IIA (NMIIA) inhibitor. Wiskostatin is a selective neural Wiskott-Aldrich syndrome protein (N-WASP) inhibitor by binding GTPase binding domain to prevent activation of the actin-polymerizing protein Arp2/3. MRCK, myotonic dystrophy kinase-related Cdc42-binding kinases. **(C)** Detection of CaHEV ORF3 RNA in the LMH^OATP1A2^ cells treated with ML141 by qPCR. ns, no significant; *P < 0.05; **P < 0.01; ***P < 0.001.

To determine the relationship between the expression of CDC42 and avian HEV infection in the cells, CDC42 was overexpressed and knocked down in the LMH^OATP1A2^ cells. After the LMH^OATP1A2^ cells were transiently transfected with pLVX-ZsGreen-CDC42 plasmids, both Immunofluorescence Assay (IFA) and Western blotting showed that the expression levels of CDC42 increased (Supplementary Figures 2A,B). Next, knockdown assays were conducted using three synthesized small interfering RNAs (siRNAs designated siCDC42-323, siCDC42-578, and siCDC42-721) that target CDC42 mRNAs and a control siRNA (siNCtrl) at a concentration of 10 nM (Table 2). The results demonstrated that the CDC42 mRNA and protein levels in LMH^OATP1A2^ cells were effectively knocked down, with the greatest effects observed in the siCDC42-721-transfected group (Supplementary Figures 2C,D). So, siCDC42-721 was used for further study, which working concentrations were determined as 10 and 40 nM (Supplementary Figures 2E,F). After CDC42 overexpression and knockdown assays in LMH^OATP1A2^ cells were developed, viral infection assays were conducted. The results showed that avian HEV RNA increased in the cells with overexpression of CDC42 and decreased in the knockdown cells (Figure 3A), indicating that the expression levels of CDC42 were positively correlated with avian HEV infection in host cells. In addition, negative-strand CaHEV RNA was also detected in these treated cells, confirming that CaHEV successfully replicated in these cells (Supplementary Figure 3).

ML141, a known selective inhibitor of CDC42, had previously been shown to suppress CDC42 activation by blocking GTP binding to CDC42 (Figure 3B; Weinberg et al., 2014). The inhibitor was used to treat the LMH^OATP1A2^ cells, and then CaHEV infected the treated cells. The maximal concentration of ML141 to treat cells was 10 μM (Supplementary Figure 4A), which was determined using a Cell Counting Kit-8 (CCK-8). Then, viral infection assays showed that the CaHEV RNA significantly decreased in the ML141-treated LMH^OATP1A2^ cells with 1 and 10 μM (Figure 3C), indicating that ML141 inhibited CaHEV infection in a dose-dependent manner.

### Cell Division Control Protein 42/RAC1-MRCK-MYLK-NMIIA Signaling Pathway Participates in Avian Hepatitis E Virus Infection

**FIGURE 4:**
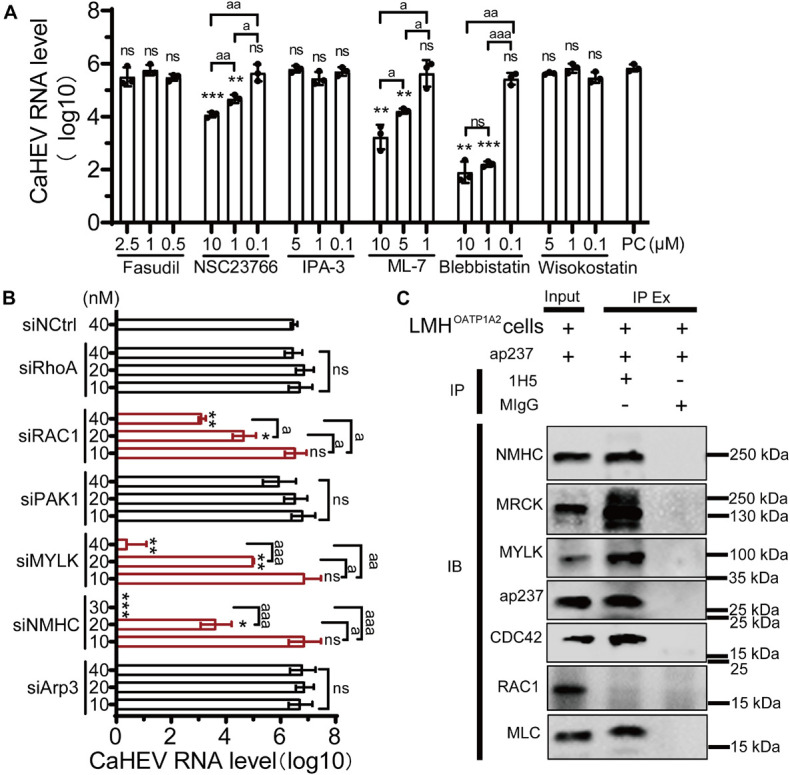
CDC42/RAC1-NMIIA signaling pathway is involved in avian infection. **(A)** Copies of CaHEV RNA in infected LMH^OATP1A2^ cells treated with different concentrations of Fasudil, NSC23766, IPA-3, ML7, wiskostatin, and blebbistatin. At 7 dpi, CaHEV ORF3 RNA was detected by qPCR. **(B)** Copies of CaHEV in infected LMH^OATP1A2^ cells transfected with different concentrations of siRhoA, siRAC1, siPAK1, siMYLK, siNMHC, and siArp3, respectively. **(C)** The ap237 pulled down the molecules in the CDC42-MRCK-MYLK-NMIIA signaling pathway. NMHC, NMIIA heavy chain. MLC, NMIIA light chain. MYLK, myosin light chain kinase. MIgG was set as blank control. ns, no significant; * and a, *P* < 0.05; ** and aa, *P* < 0.01; *** and aaa, *P* < 0.001.

CDC42 is a key molecule within cellular cytoskeleton-associated Rho GTPase signaling pathways, and some viruses can exploit these pathways to participate in the life cycle of viral replication (Farhan and Hsu, 2016). Importantly, molecular pathways downstream of cytoskeleton-associated signaling pathways play diverse and complicated roles during viral infection by engaging in interactions with a high degree of overlap, crosstalk, and dynamic characteristics (Swaine and Dittmar, 2015; Trejo-Cerro et al., 2019). To confirm which pathways were involved in avian HEV infection, six well-characterized inhibitors of the Rho GTPases family were used, including Fasudil for Rho-associated protein kinase (ROCK), NSC23766 for RAC1, IPA-3 for p21-activated kinase 1 (PAK1), ML7 for myosin light chain kinase (MLCK), wiskostatin for neural Wiskott-Aldrich syndrome proteins (N-WASP), and blebbistatin for non-muscle myosin IIA (NMIIA) (Figure 3B). The optimized inhibitors concentrations were first determined to minimize cytotoxicity toward LMH^OATP1A2^ cells (Supplementary Figure 4B). After optimal inhibitor concentrations were used to treat the cells, the viral infection assay was performed. The results showed that the amounts of CaHEV RNA in the LMH^OATP1A2^ cells treated with inhibitors NSC23766, ML7, and blebbistatin were significantly less than the levels observed for controls and the cells treated with other inhibitors (Figure 4A). Next, the knockdown assays were conducted using six siRNAs that target the mRNAs of RhoA, RAC1, PAK1, MYLK, non-myosin heavy chain (NMHC), and Arp3, respectively (siRNAs designated siRhoA, siRAC1, siPAK1, siMYLK, siNMHC, and siArp3) (Table 2). The results showed that the six proteins in LMH^OATP1A2^ cells were effectively knocked down (Supplementary Figure 5). Then, viral infection assays were carried out after the knockdown assay in LMH^OATP1A2^ cells was developed. The result showed that avian HEV RNA significantly decreased in the LMH^OATP1A2^ cells transfecting with siRAC1, siMYLK, and siNMHC in a dose-dependent manner (Figure 4B). These results indicated that CDC42/RAC1-MRCK-MYLK-NMIIA signaling pathway was involved in avian HEV infection in host cells. It has been reported that the upstream components of the Rho GTPases family activate its downstream components by binding with each other (Manser et al., 1994; Leung et al., 1998; Clayburgh et al., 2004; Vicente-Manzanares et al., 2009). To further confirm the involvement of this signaling pathway in CaHEV infection, the pull-down assays revealed that the members of the CDC42-MRCK-MYLK-NMIIA signaling pathway were pulled down by ap237, while RAC1 was not (Figure 4C). The results further confirmed that the CDC42-MRCK-MYLK-NMIIA signaling pathway was associated with avian HEV infection in the cells.

### Cell Division Control Protein 42 Interacts With Mammalian Hepatitis E Virus Capsid Protein and Participates in Viral Infection

**FIGURE 5:**
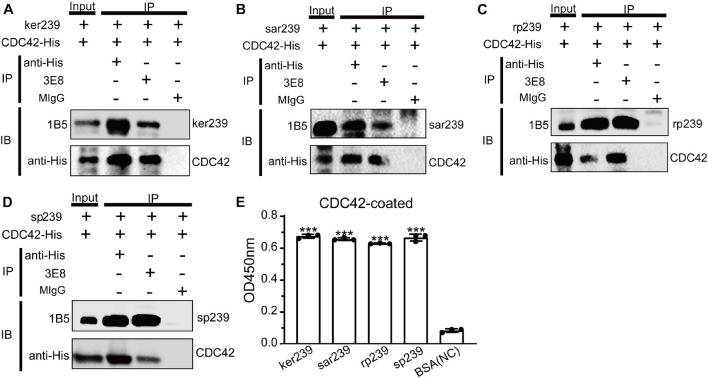
CDC42 interacts with truncated capsid proteins of mammalian HEV. It was pulled down from each other using 3E8 mAb or anti-His mAb that CDC42-His and ker239 from genotype 3 HEV Kernow C1/p6 strain (HEV-3) **(A)**, sar239 from genotype 1 HEV Sar55 strain **(B)**, sp239 from the genotype 4 HEV CHN-SD-sHEV strain **(C)**, and rp239 from genotypes 3 HEV CHN-SX-rHEV strain **(D)**. MIgG was set as a blank control. Out of 600 μL of total proteins, 40 μL was set as the input group, and the additional proteins were trisected and used for pull-down assays. **(E)** ELISA results of CDC42-His directly binding with different HEV capsid proteins. Plates were coated with CDC42-His and incubated with ker239, sar239, rp239, and sp239. ***P < 0.001.

Sequences alignments showed that CDC42 is highly conserved among diverse species (Supplementary Figure 6). Genotype 1 and 3 human HEVs (Sar55 and Kernow-C1/p6, respectively), genotype 4 swine HEV (CHN-SD-sHEV), and genotype 3 rabbit HEV (CHN-SX-rHEV) were evaluated using assays as described above for avian HEV to determine whether CDC42 also participates in mammalian HEV infection. Four truncated capsid proteins of the abovementioned mammalian HEVs (sar239, ker239, sp239, and rp239) were used to determine the interactions with CDC42. The results of Co-IP and ELISA showed that all four capsid proteins are directly bound to CDC42 (Figure 5). These results suggested that the CDC42 may directly interact with mammalian HEV capsid proteins, as observed for ap237.

**FIGURE 6:**
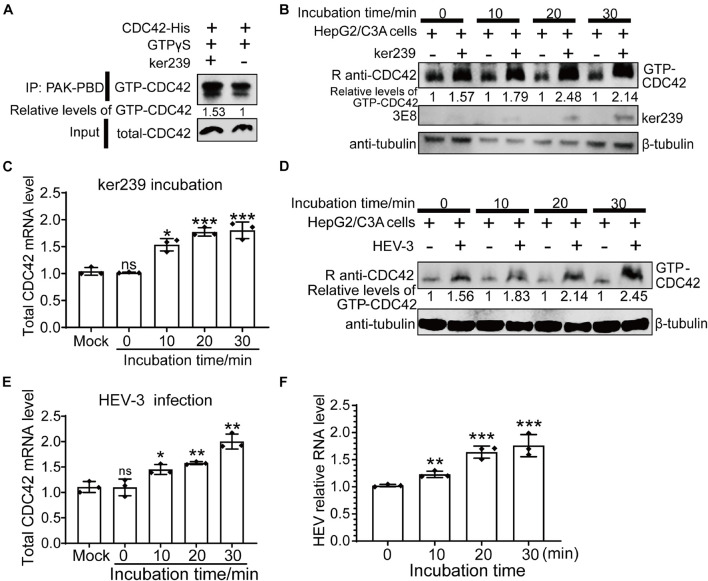
Upregulation of expression levels of GTP-CDC42 and total CDC42 in ker239 treated and non-enveloped HEV infected cells. **(A)** CDC42 binding more GTPs through interaction with ker239 *in vitro*. **(B)** Expression levels of GTP-CDC42 in HepG2/C3A cells treated with ker239 at 0, 10, 20, and 30 min. **(C)** Total mRNA levels of CDC42 in HepG2/C3A cells treated with ker239 by qPCR. **(D)** Expression levels of GTP-CDC42 in HepG2/C3A cells inoculated with naked HEV-3 at 0, 10, 20, and 30 min-post-inoculation. **(E,F)** The mRNA levels of total CDC42 **(E)** and HEV-3 RNA **(F)** in HepG2/C3A cells inoculated with HEV-3 by qPCR. RNA levels were normalized to human GAPDH. ns, no significant; *P < 0.05; **P < 0.01; ***P < 0.001.

Although avian HEV capsid protein shares 48–50% amino acid identity with mammalian HEV, there is a high degree (>90%) of identity among human, swine, and rabbit HEV capsid proteins (Haqshenas et al., 2001). In addition, because only the HEV strain Kernow-C1/p6 (HEV-3) can be efficiently propagated *in vitro*, ker239 and Kernow-C1/p6 were selected to study mammalian HEV infection in host cells. Firstly, CDC42 activation assays *in vitro* demonstrated that the interaction between CDC42 and ker239 also facilitated CDC42 binding to more GTPs *in vitro* (Figure 6A). The protein levels of GTP-CDC42 and mRNA of total CDC42 also increased gradually if the HepG2/C3A cells were pretreated with ker239 for 10, 20, and 30 min (Figures 6B,C). For virus infection assay, non-enveloped HEV-3 virions were first produced by removing the viral envelope of HEV-3 virions from the HEV-positive culture supernatant and identified by iodixanol density gradient centrifugation based on previously mentioned information reported methods (Nagashima et al., 2014; Yin et al., 2016). The density of the viral particles was found to range from 1.177 to 1.261 g/mL, indicating that viral envelopes were removed successfully (Supplementary Figure 7A). After non-enveloped HEV-3 was inoculated, the protein levels of GTP-CDC42 and mRNA of total CDC42 also increased gradually in the HepG2/C3A cells (Figures 6D,E). Meanwhile, HEV-3 RNA was also increased, confirming that HEV-3 successfully infected the HepG2/C3A cells (Figure 6F). These results indicated that HEV infection could promote the up-regulation of CDC42 expression in the HepG2/C3A cells.

**FIGURE 7:**
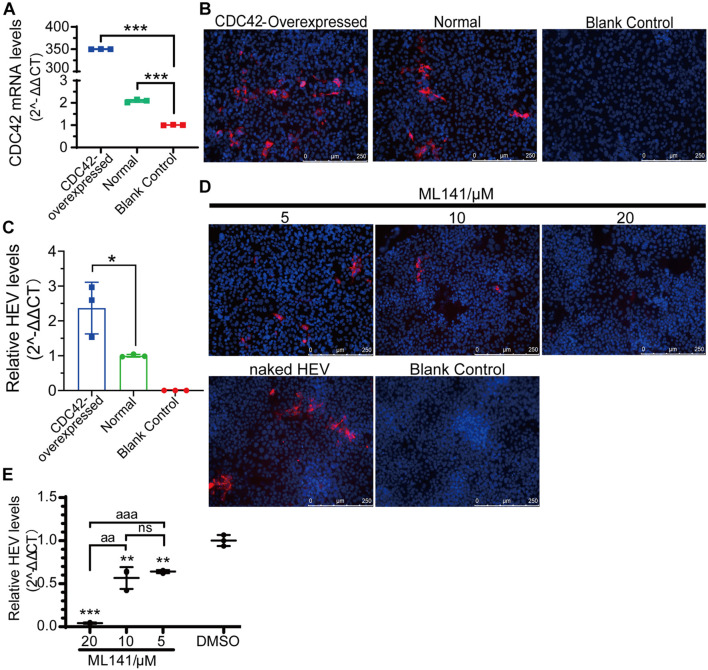
Positive correlation between expression level and activity of CDC42 and naked HEV-3 infection in HepG2/C3A cells. **(A)** Relative CDC42 mRNA levels in HEV-infected HepG2/C3A cells with different pretreatment. Increasing of HEV-3 infection in the CDC42-overexpressed HepG2/C3A cells by IFA **(B)** and qPCR detection **(C)**. IFA **(D)** and qPCR **(E)** detect non-enveloped HEV-3 in ML141-pretreated HepG2/C3A cells. The result of the qPCR assay was normalized to human GAPDH. ns, no significant; *, *P* < 0.05; ** and aa, *P* < 0.01; *** and aaa, *P* < 0.001.

To further confirm the relationship between CDC42 expression and HEV-3 infection, overexpression of CDC42 following viral infection was performed. The results showed that the mRNA of CDC42 increased after the HepG2/C3A cells were transfected with the plasmids, indicating that the CDC42 was overexpressed (Figure 7A). The IFA results showed that the red fluorescence (HEV capsid proteins) significantly increased in the overexpressed cells (Figure 7B). In addition, after viral infection, the amounts of HEV RNA in the overexpressed-CDC42 group were more than that of the normal group (Figure 7C). These results indicated that overexpression of CDC42 in the HepG2/C3A cells could promote HEV-3 infection.

Moreover, the ML141 inhibitor was also used to analyze the relationship. The optimized concentrations of ML141 were firstly determined for the HepG2/C3A cells, and the maximal concentration was 20 μM (Supplementary Figure 7C). Then, the IFA and qPCR results of viral infection showed that the HEV protein and RNA were both decreased in a dose-dependent manner after the HepG2/C3A cells were treated with 5, 10, and 20 μM of ML141 (Figures 7D,E). Thus, these results collectively suggested that CDC42 is also potentially associated with the mammalian HEV infection.

### Different Cell Division Control Protein 42 Downstream Pathways Are Involved in Non- and Quasi-Enveloped Hepatitis E Virus Infection

**FIGURE 8:**
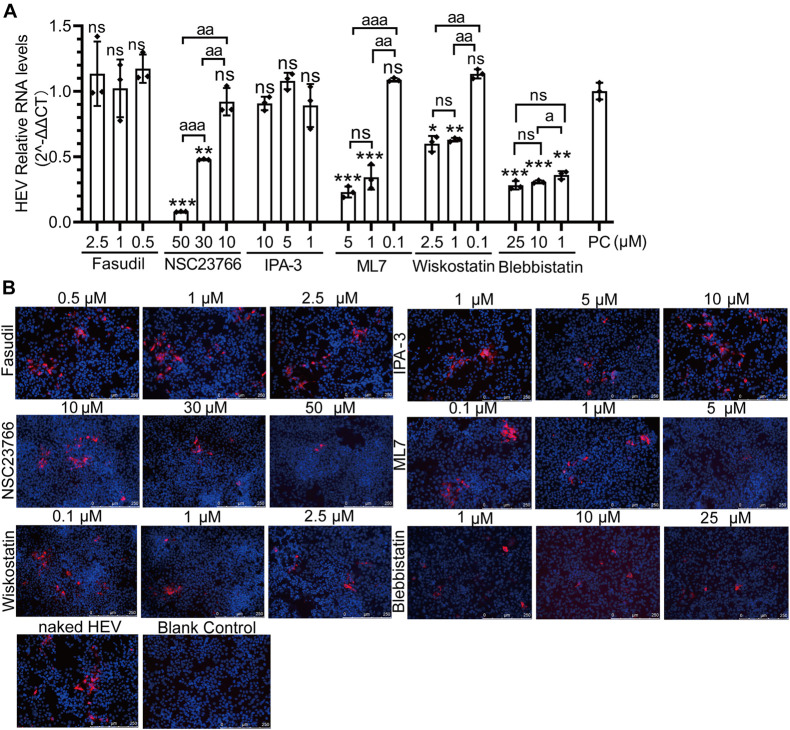
CDC42/RAC1-MRCK-MYLK-NMIIA and CDC42/RAC1-(N-) WASP-Arp2/3 signaling pathways participate in non-enveloped HEV infection in HepG2/C3A cells. Relative mRNA levels **(A)** and capsid protein levels **(B)** of non-enveloped HEV-3 at 5 dpi in HepG2/C3A cells treated with Fasudil, NSC23766, IPA-3, ML7, wiskostatin, and blebbistatin. The results were normalized with GAPDH mRNA in each group. The capsid protein (red) location was analyzed, and the nucleus indicated DAPI (blue) staining in the images. Scale bars, 250 μm. ns, no significant; * and a, *P* < 0.05; ** and aa, *P* < 0.01; *** and aaa, *P* < 0.001.

There are two forms of natural HEV particles, non-enveloped HEV in bile and feces and quasi-enveloped HEV in blood and cell culture supernatants (Feng et al., 2013; Yin et al., 2016; Nagashima et al., 2017; Himmelsbach et al., 2018; Rivera-Serrano et al., 2019; Oechslin et al., 2020; Tallan and Feng, 2020). The other six inhibitors of the Rho GTPases family (Fasudil, NSC23766, IPA-3, ML7, wiskostatin, and blebbistatin) were used to demonstrate the correlation between the two forms of HEV infection and CDC42 downstream signaling pathways. Firstly, the concentrations of these inhibitors were optimized for treating HepG2/C3A cells (Supplementary Figure 7D). Then, three concentrations of each inhibitor showing no toxicity for the cells were selected. After the pretreated cells were inoculated with non-enveloped HEV, the qPCR results showed that the HEV RNA was decreased in a dose-dependent manner in the NSC23766, ML7, wiskostatin, and blebbistatin treated groups (Figure 8A). The IFA results also showed that the red fluorescence decreased in these groups (Figure 8B). These results indicated that the NSC23766, ML7, wiskostatin, and blebbistatin could inhibit non-enveloped HEV infection. Based on the signaling pathways inhibited by the inhibitors, we think that CDC42/RAC1-MRCK-MYLK-NMIIA and CDC42/RAC1-(N-) WASP-Arp2/3 signaling pathways are involved in non-enveloped HEV infection.

**FIGURE 9:**
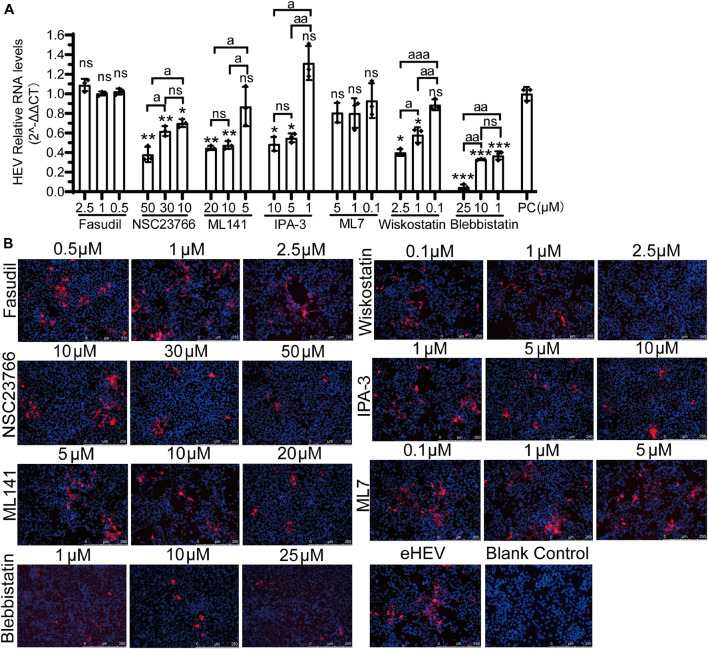
CDC42/RAC1-PAK1 and CDC42/RAC1-(N-) WASP signaling pathways participate in quasi-enveloped HEV infection in HepG2/C3A cells. **(A)** There are relative mRNA levels of quasi-enveloped HEV-3 in HepG2/C3A cells treated with Fasudil, NSC23766, ML141, IPA-3, ML7, wiskostatin, and blebbistatin. The results were normalized with GAPDH mRNA in each group. **(B)** A detection of quasi-enveloped HEV-3 (designated eHEV) in HepG2/C3A cells at 5 dpi. Anti-HEV-ORF2 antibody (red) was used to detect the virus. Nuclei were counterstained with DAPI (blue). Scale bars, 250 μm. ns, no significant; * and a, *P* < 0.05; ** and aa, *P* < 0.01; *** and aaa, *P* < 0.001.

Previously, it was reported that the life cycles of non- and quasi-enveloped HEV infection in the host cells are different. Then, these inhibitors were also used to analyze quasi-enveloped HEV infection assays. Firstly, the quasi-enveloped HEV virions were also produced and confirmed that the density of quasi-enveloped HEV particles was range from 1.070 to 1.139 g/mL (Figure 9B), which was consistent with the records in other literature (Nagashima et al., 2014; Yin et al., 2016). After the cells were treated with three concentrations of each inhibitor and inoculated with the quasi-enveloped HEV, the results of qPCR and IFA showed that the HEV RNA and protein were decreased in a dose-dependent manner in the groups of ML141, NSC23766, IPA-3, wiskostatin, and blebbistatin (Figure 9). These results indicated that these inhibitors inhibited quasi-enveloped HEV infection in HepG2/C3A cells. Similarly, based on the signaling pathways inhibited by these inhibitors, we speculate that the CDC42/RAC1-PAK1-NMIIA/cofilin and CDC42/RAC1-(N-) WASP-Arp2/3 signaling pathways were involved in quasi-enveloped HEV infection.

## Discussion

Due to the lack of highly effective culture systems *in vitro*, the life cycle of HEV remains unclear. Using HEV-like particles (HEV-LPs), several host factors have been determined to participate in naked HEV infection, including cell attachment, entry, and/or trafficking (Kamar et al., 2009; Yu et al., 2011; Kapur et al., 2012; Holla et al., 2015; Ahmed et al., 2016; Zhang et al., 2016; Li et al., 2019; Shiota et al., 2019). For example, during clathrin- and dynamin-dependent endocytosis, membrane cholesterol, the PI3K pathway, and actin have been shown to participate in cell entry and membrane trafficking of naked HEV particles (Kapur et al., 2012; Holla et al., 2015). The present study confirmed that the host factor CDC42 directly interacted with human, swine, rabbit, and avian HEV capsid proteins, facilitating CDC42 binding to more GTPs. Hence, our results further confirmed that HEV-LPs could be used with natural naked viral particles to investigate virus-host interaction. Moreover, HEV infection could upregulate the amounts of total CDC42 and GTP-CDC42 in the host cells, which activates CDC42 sub-pathways. Using the different inhibitors of CDC42 sub-pathways, the results showed that the CDC42/RAC1-MRCK-MYLK-NMIIA pathway was involved in naked HEV infection in the host cells (Figure 4). Intriguingly, in the present study, RAC1, another member of the Rho GTPases family, was also involved in non-enveloped HEV infection according to the result of CaHEV infection assay in the NSC23766-pretreated LMH^OATP1A2^ cells (Figure 4A). However, RAC1 was not pulled down by ap237, indicating that the avian HEV capsid protein does not directly interact with RAC1 (Figure 4B). The results suggested that non-enveloped HEV may indirectly trigger the RAC1 signaling pathway being involved in the viral infection as observed for other viruses (Krautkramer et al., 2004; Krzyzaniak et al., 2013; Swaine and Dittmar, 2015; Dun et al., 2020; Kolenda et al., 2020). Meanwhile, these results also suggested that different signaling pathways and ways are involved in non-enveloped HEV infection.

Some previous studies have documented that CDC42 signaling pathways can be involved in different stages of viral infection, including viral entry, intracellular trafficking, egress, and cell-to-cell transmission. For example, porcine reproductive and respiratory syndrome virus, porcine hemagglutinating encephalomyelitis virus, and equine alpha herpesvirus used these pathways to facilitate them entering into the host cells (Lv et al., 2018; Kolyvushko et al., 2020; Wei et al., 2020). Besides that, Japanese encephalitis virus, pseudorabies virus, herpes simplex virus, Ebola virus, human immunodeficiency virus, and Epstein-Barr virus hijacked these pathways to participate in viral intracellular trafficking, egress, and cell-to-cell transmission (Nikolic et al., 2011; Lu et al., 2013; Do et al., 2014; Yu et al., 2019; Jansens et al., 2020). Here, we found that CDC42 directly interacts with HEV capsid protein and is involved in HEV infection in the host cells. However, as we have known, because HEV enters into the cells through endocytosis and its capsid is not exposed to the cytosol, we speculate that CDC42 may be involved in intracellular trafficking and egress stages of HEV infection. After HEV capsid proteins were expressed in the cytoplasm for viral assemble, they directly interact with CDC42 following viral infection.

The present study found that both naked avian and mammalian HEV used the CDC42-MRCK pathway, quasi-enveloped HEV used the CDC42-PAK1 pathway, and all HEV virions used the CDC42-(N-)WASP pathway. These findings indicated that non- and quasi-enveloped HEVs hijack different CDC42 sub-pathways to be involved in viral infection. Previously, it was reported that the entering stage of non- and quasi-enveloped HEV is different, and the other stages in the cytoplasm are the same (Holla et al., 2015; Yin et al., 2016). So, we speculate that the CDC42-(N-)WASP pathway may be involved in the intracellular trafficking and egress stages of HEV infection. Moreover, the CDC42-MRCK pathway may participate in the entry of naked HEV, and the CDC42-PAK1 pathway could potentially enter quasi-enveloped HEV. These speculations may be determined in the future through viral endocytosis experiments.

As to the above speculations, the interaction between CDC42 and HEV capsid protein may occur in the stages of viral intracellular trafficking and egress. So, we think that the CDC42-(N-)WASP pathway may be activated through the interaction. However, the CDC42-MRCK and CDC42-PAK1 pathway being involved in the entry of HEV may be activated in other ways. Based on some previous studies, many enveloped virions can bind to T-cell immunoglobulin and mucin (TIM) domains and induce micropinocytosis through activation of Rho GTPase signaling pathways. Then, the pathways induce endocytosis after host receptors bind to viral envelope phosphatidylserine groups (Do et al., 2014; Yu et al., 2019; Kolyvushko et al., 2020). Thus, quasi-enveloped HEV particles may interact with TIM to induce micropinocytosis *via* CDC42 signaling pathways or indirectly trigger CDC42 signaling pathways to facilitate viral entry. Altogether, our study indicated that CDC42 signaling pathways are involved in non-enveloped and quasi-enveloped HEV infection, and HEV may exploit several host CDC42 signaling pathways to participate in different stages of HEV infection.

## Conclusion

In summary, this study revealed that CDC42 directly interacts with HEV capsid proteins, which facilitates CDC42 binding to more GTP. Subsequently, a positive correlation between the expression and activity of CDC42 and HEV infection was determined. In addition, the different CDC42 downstream pathways were triggered based on particle forms of HEV. The CDC42-MRCK-NMIIA pathway was involved in non-enveloped HEV infection, the CDC42-PAK1-NMIIA/Cofilin pathway was involved in quasi-enveloped HEV infection, and the CDC42-(N-) WASP-Arp2/3 pathway was involved in both two forms of HEV infection. These results provide new insights into the HEV infection in the host cells and guide the development of novel therapeutic targets the control HEV infection.

## Data Availability Statement

The original contributions presented in the study are included in the article/Supplementary Material, further inquiries can be directed to the corresponding author/s.

## Author Contributions

E-MZ, QZ, and JH conceived the study. MF performed the research, analyzed data, and drafted the manuscript. YL, BZ, and JW contributed to the construction of plasmids and ELISA assays. BL and TC contributed to the animal study. YS and YN contributed to the cell culture. JH, QZ, and E-MZ revised and finalized the manuscript. All authors contributed to the article and approved the submitted version.

## Conflict of Interest

The authors declare that the research was conducted in the absence of any commercial or financial relationships that could be construed as a potential conflict of interest.

## Publisher’s Note

All claims expressed in this article are solely those of the authors and do not necessarily represent those of their affiliated organizations, or those of the publisher, the editors and the reviewers. Any product that may be evaluated in this article, or claim that may be made by its manufacturer, is not guaranteed or endorsed by the publisher.

## Supplementary Material

Supplementary Figure 1 Co-localization of ap237 and CDC42 in HEK 293T cells. **(A)** SDS-PAGE analysis of purified ap237 and CDC42-His protein. **(B)** ap237 co-located with CDC42 in HEK 293T cells. The location of CDC42 (green) and ap237 (red) was analyzed by confocal microscopy. Nuclei were counterstained with DAPI (blue). Outline regions are magnified (inset) **(A)**, and profiles of fluorescence intensity along the yellow line in corresponding images are shown in right panels analyzed using Image J software **(B)**.

Supplementary Figure 2 Expression levels of CDC42 in treated cells. CDC42 overexpression in the LMH^OATP1A2^ cells was analyzed using fluorescence microscopy **(A)** and Western blotting **(B)**. BC, blank control, means untreated normal LMH^OATP1A2^ cells. Scale bar, 100 μm. Relative mRNA levels **(C)** and protein levels **(D)** of CDC42 in LMH^OATP1A2^ cells transfected with three CDC42-specific siRNAs (siCDC42-323, siCDC42-578 and siCDC42-721) or a control siRNA (siNCtrl). Detection of CDC42 expression in LMH^OATP1A2^ cells transfected with different concentrations (10, 20, and 40 nM) of siCDC42-721 by qPCR **(E)** and Western blotting **(F)**.

Supplementary Figure 3 Positive correlations between expression levels of CDC42 and amount of CaHEV entering cells. Negative-strand RNA of CaHEV was detected by nested RT-PCR, and the size was approximately 250 bp. Blank control means the group of CaHEV-uninoculated LMH^OATP1A2^ cells; Extraction Control means the blank control on RNA extraction step; Round 1 and Round 2 control are the blank controls on Round 1 PCR and Round 2 PCR step, respectively.

Supplementary Figure 4 Cytotoxicity analysis of Rho family GTPases inhibitors in LMH^OATP1A2^ cells. **(A)** Cytocidal assay was performed using the CCK-8 kit on ML141-treated LMH^OATP1A2^ cells. **(B)** Cytotoxicity of Fasudil, NSC23766, IPA-3, Wiskostatin, ML7, and Blebbistatin in LMH^OATP1A2^ cells.

Supplementary Figure 5 Expression levels of the proteins of Rho GTPase family in siRNA-transfected LMH^OATP1A2^ cells. Protein levels of RhoA **(A)**, RAC1 **(B)**, PAK1 **(C)**, MYLK **(D)**, non-myosin heavy chain (NMHC) **(E)** and Arp3 **(F)** in LMH^OATP1A2^ cells transfected with different concentrations [10, 20, and 40 nM (or 30 nM)] of siRhoA, siRAC1, siPAK1, siMYLK, siNMHC and siArp3, respectively.

Supplementary Figure 6 Amino acid alignments of CDC42 from different species. The Clustal W module of the MegAlign program of Lasergene 7.1 (DNASTAR Inc., MI, United States) was used.

Supplementary Figure 7 Identification of HEV virions and cytotoxicity analysis of Rho family GTPases inhibitors in HepG2/C3A cells. Iodixanol gradient of non-enveloped HEV virions **(A)** and quasi-enveloped HEV virions **(B)**. Iodixanol gradients of 10–50% w/v of HEV virions. HEV RNA infraction was determined by qPCR. Cytotoxicity analysis of Rho family GTPases inhibitors in HepG2/C3A cells **(C,D)**.
