# Supplementary figures and images for: Cell Division Control Protein 42 Interacts With Hepatitis E Virus Capsid Protein and Participates in Hepatitis E Virus Infection
Source: Front Microbiol. 2021 Nov 1;12:775083. doi: 10.3389/fmicb.2021.775083 (PMC8591454; doi:10.3389/fmicb.2021.775083)

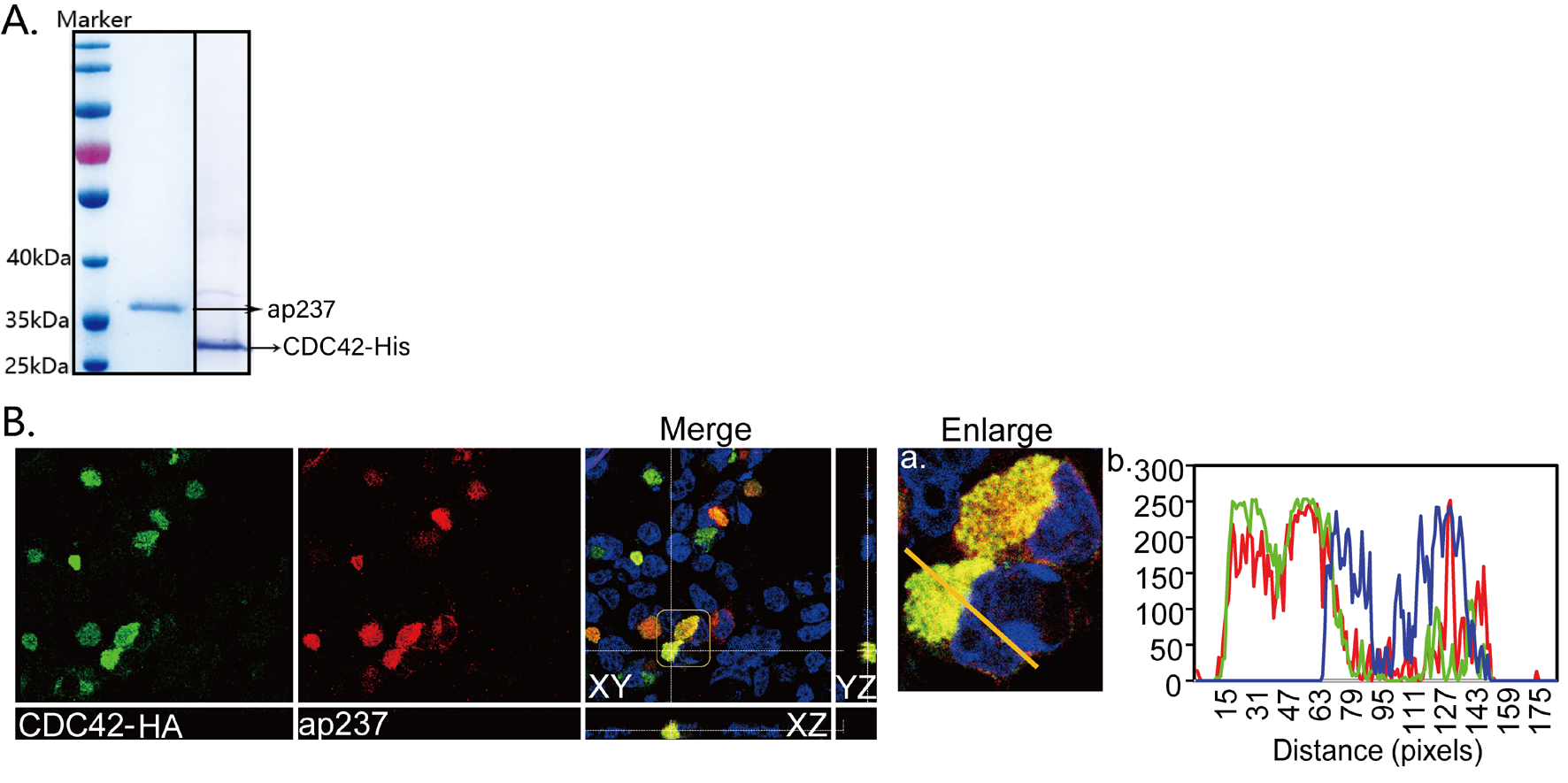

Supplement: Supplementary Figure 1 — Co-localization of ap237 and CDC42 in HEK 293T cells. (A) SDS-PAGE analysis of purified ap237 and CDC42-His protein. (B) ap237 co-located with CDC42 in HEK 293T cells. The location of CDC42 (green) and ap237 (red) was analyzed by confocal microscopy. Nuclei were counterstained with DAPI (blue). Outline regions are magnified (inset) (A), and profiles of fluorescence intensity along the yellow line in corresponding images are shown in right panels analyzed using Image J software (B). [file Image_1.TIF]

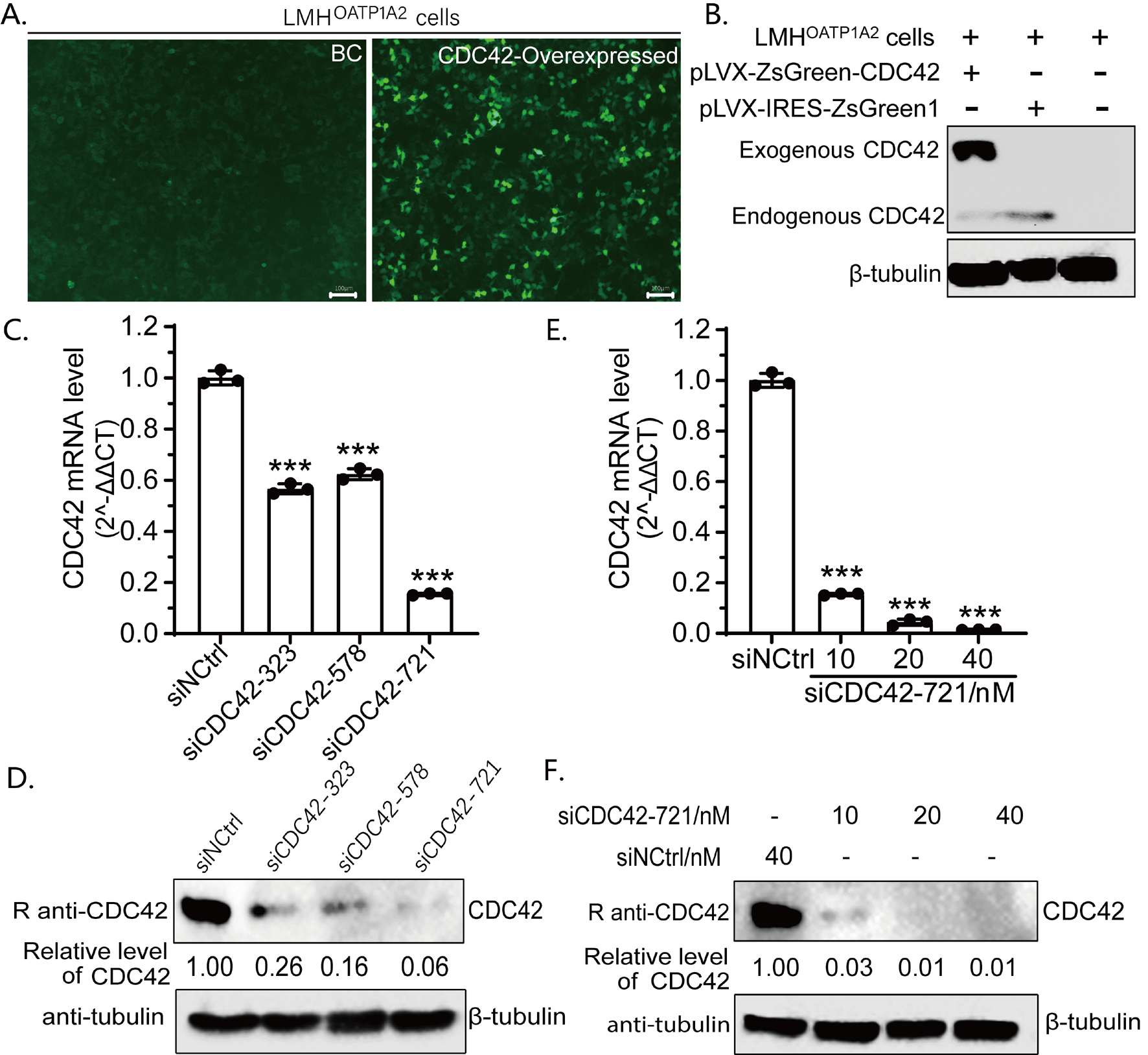

Supplement: Supplementary Figure 2 — Expression levels of CDC42 in treated cells. CDC42 overexpression in the LMHOATP1A2 cells was analyzed using fluorescence microscopy (A) and Western blotting (B). BC, blank control, means untreated normal LMHOATP1A2 cells. Scale bar, 100 μm. Relative mRNA levels (C) and protein levels (D) of CDC42 in LMHOATP1A2 cells transfected with three CDC42-specific siRNAs (siCDC42-323, siCDC42-578 and siCDC42-721) or a control siRNA (siNCtrl). Detection of CDC42 expression in LMHOATP1A2 cells transfected with different concentrations (10, 20, and 40 nM) of siCDC42-721 by qPCR (E) and Western blotting (F). [file Image_2.TIF]

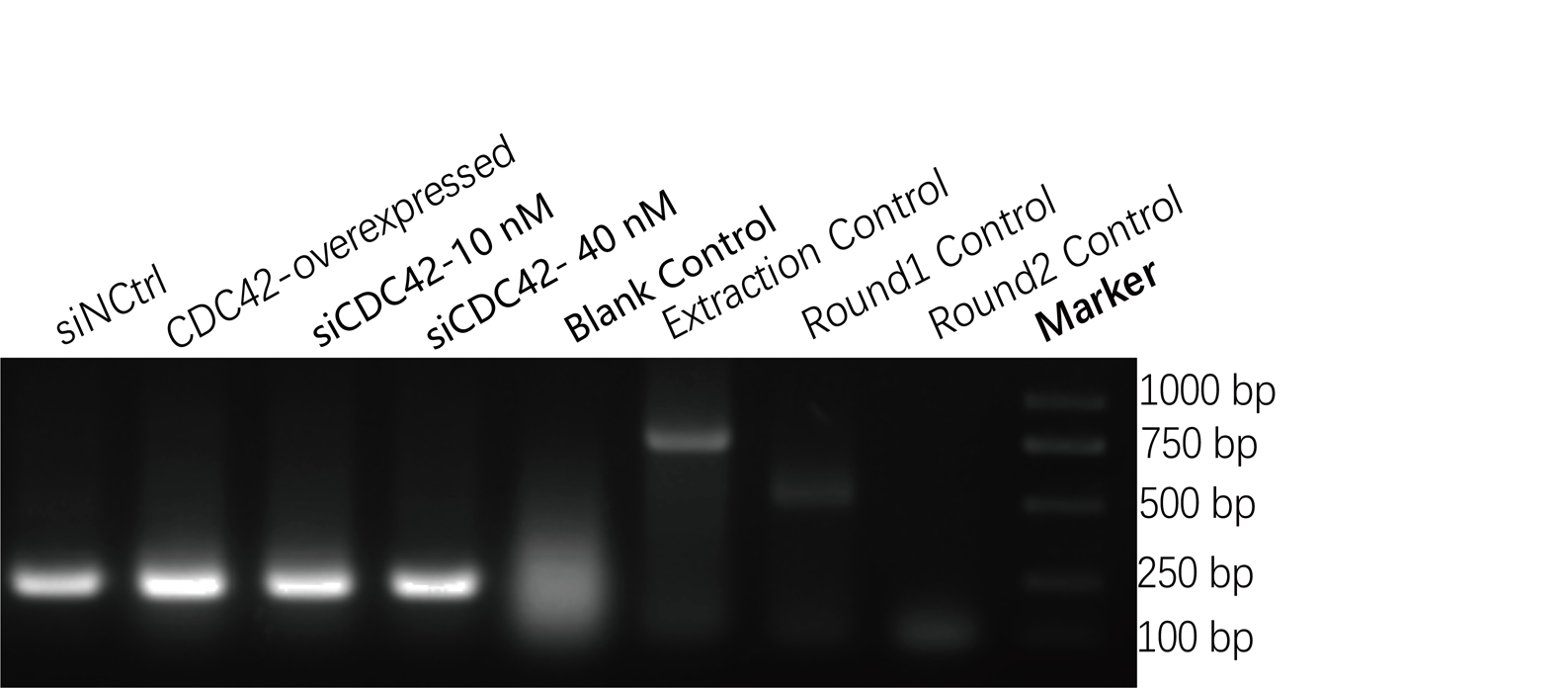

Supplement: Supplementary Figure 3 — Positive correlations between expression levels of CDC42 and amount of CaHEV entering cells. Negative-strand RNA of CaHEV was detected by nested RT-PCR, and the size was approximately 250 bp. Blank control means the group of CaHEV-uninoculated LMHOATP1A2 cells; Extraction Control means the blank control on RNA extraction step; Round 1 and Round 2 control are the blank controls on Round 1 PCR and Round 2 PCR step, respectively. [file Image_3.TIF]

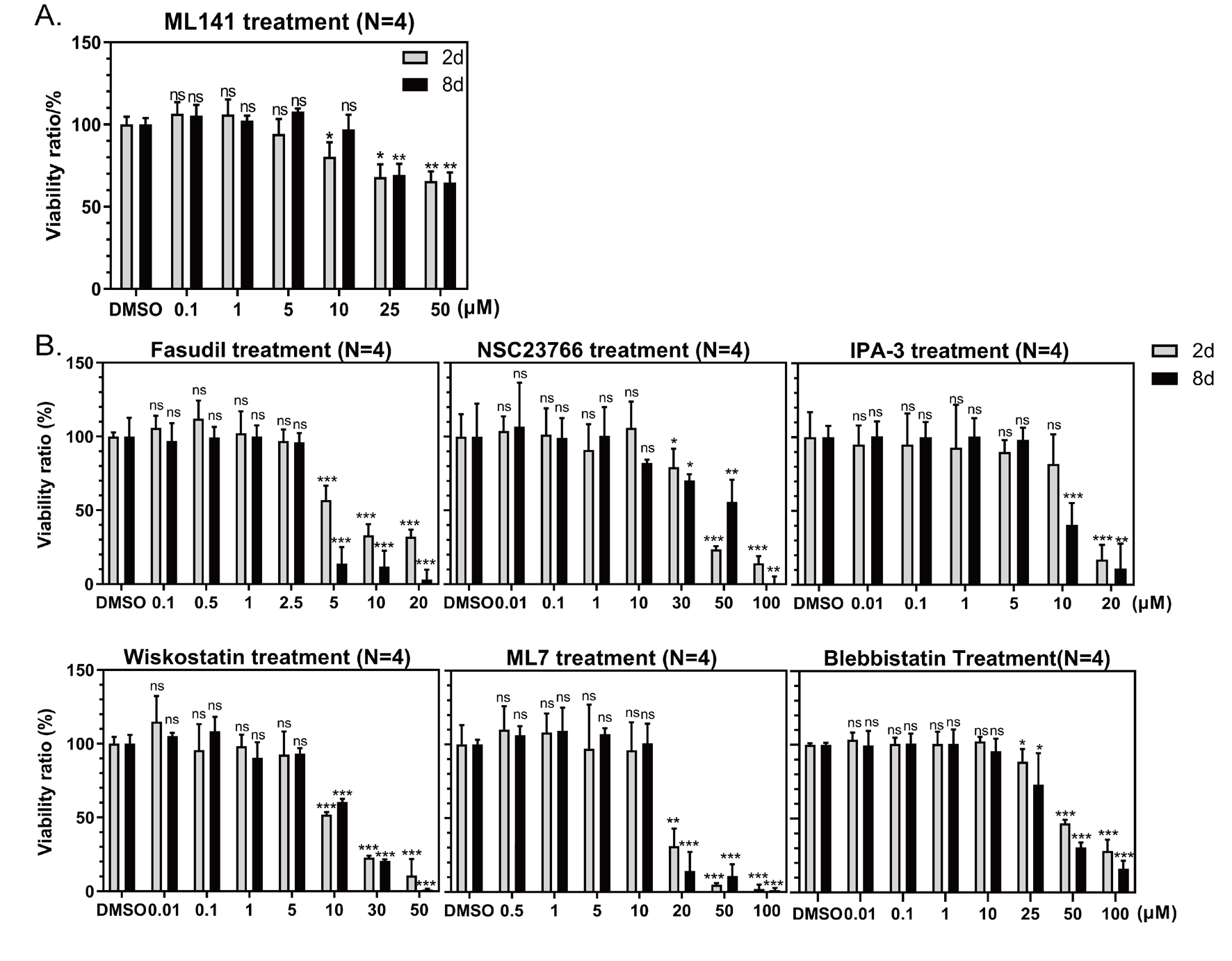

Supplement: Supplementary Figure 4 — Cytotoxicity analysis of Rho family GTPases inhibitors in LMHOATP1A2 cells. (A) Cytocidal assay was performed using the CCK-8 kit on ML141-treated LMHOATP1A2 cells. (B) Cytotoxicity of Fasudil, NSC23766, IPA-3, Wiskostatin, ML7, and Blebbistatin in LMHOATP1A2 cells. [file Image_4.TIF]

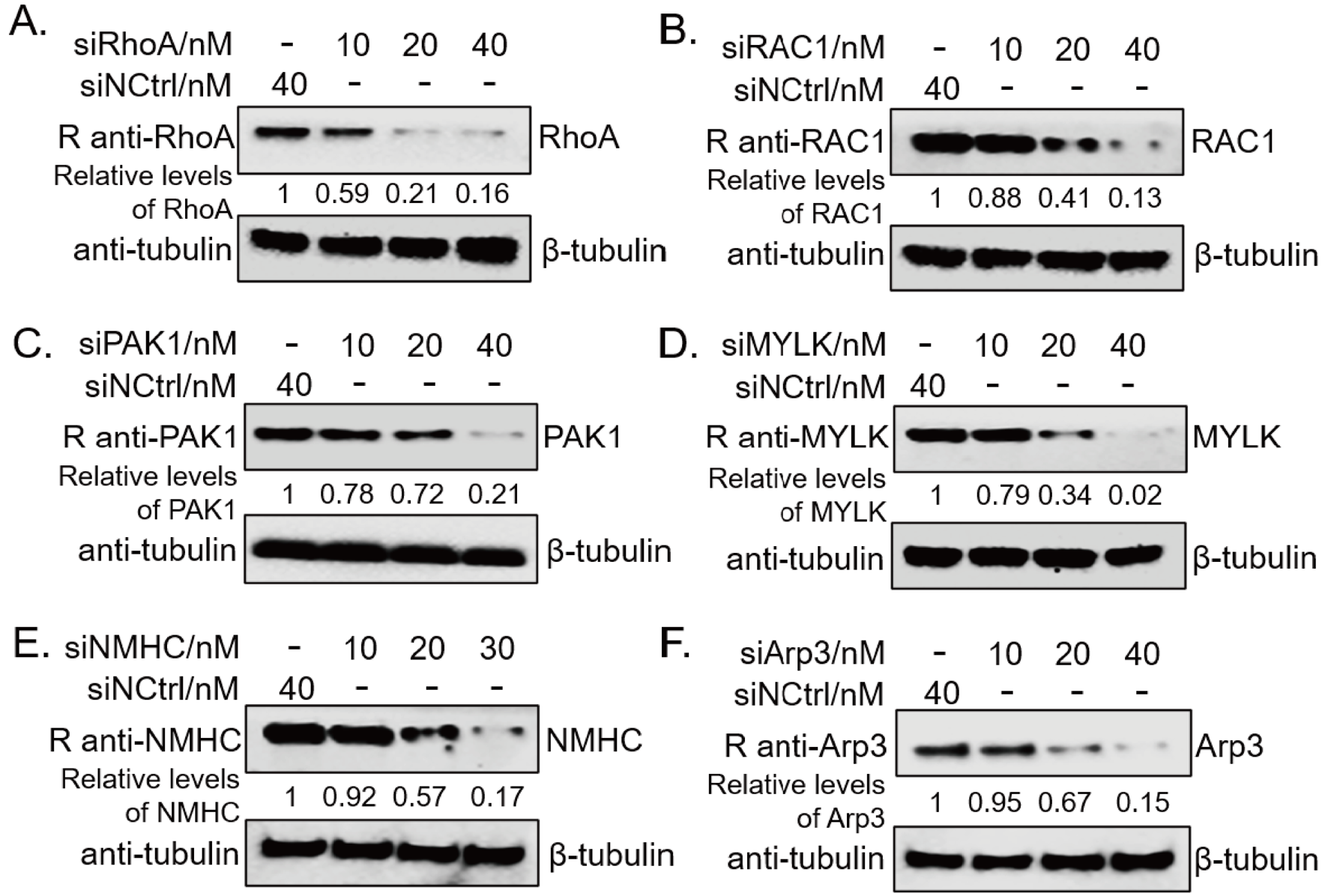

Supplement: Supplementary Figure 5 — Expression levels of the proteins of Rho GTPase family in siRNA-transfected LMHOATP1A2 cells. Protein levels of RhoA (A), RAC1 (B), PAK1 (C), MYLK (D), non-myosin heavy chain (NMHC) (E) and Arp3 (F) in LMHOATP1A2 cells transfected with different concentrations [10, 20, and 40 nM (or 30 nM)] of siRhoA, siRAC1, siPAK1, siMYLK, siNMHC and siArp3, respectively. [file Image_5.TIF]

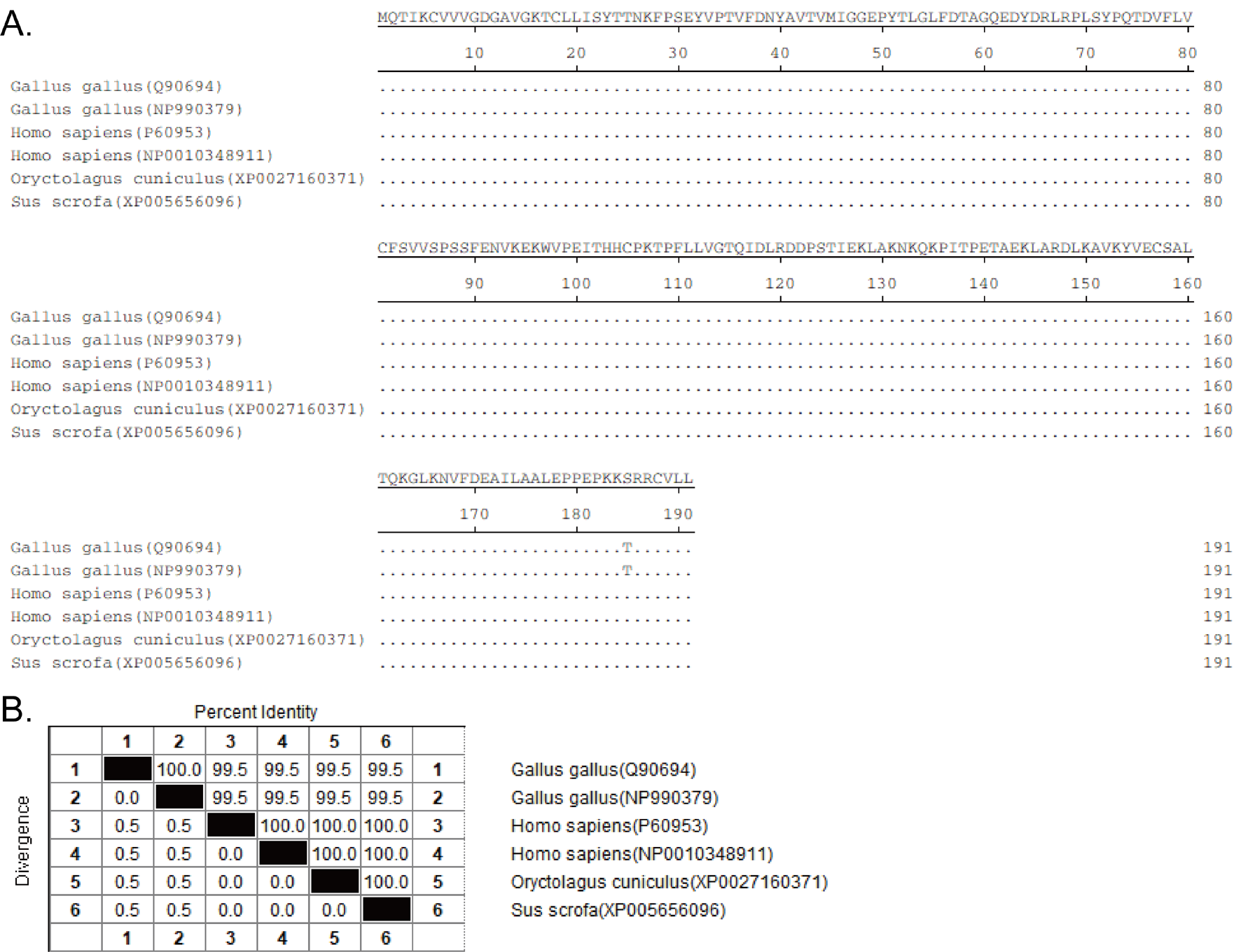

Supplement: Supplementary Figure 6 — Amino acid alignments of CDC42 from different species. The Clustal W module of the MegAlign program of Lasergene 7.1 (DNASTAR Inc., MI, United States) was used. [file Image_6.tif]

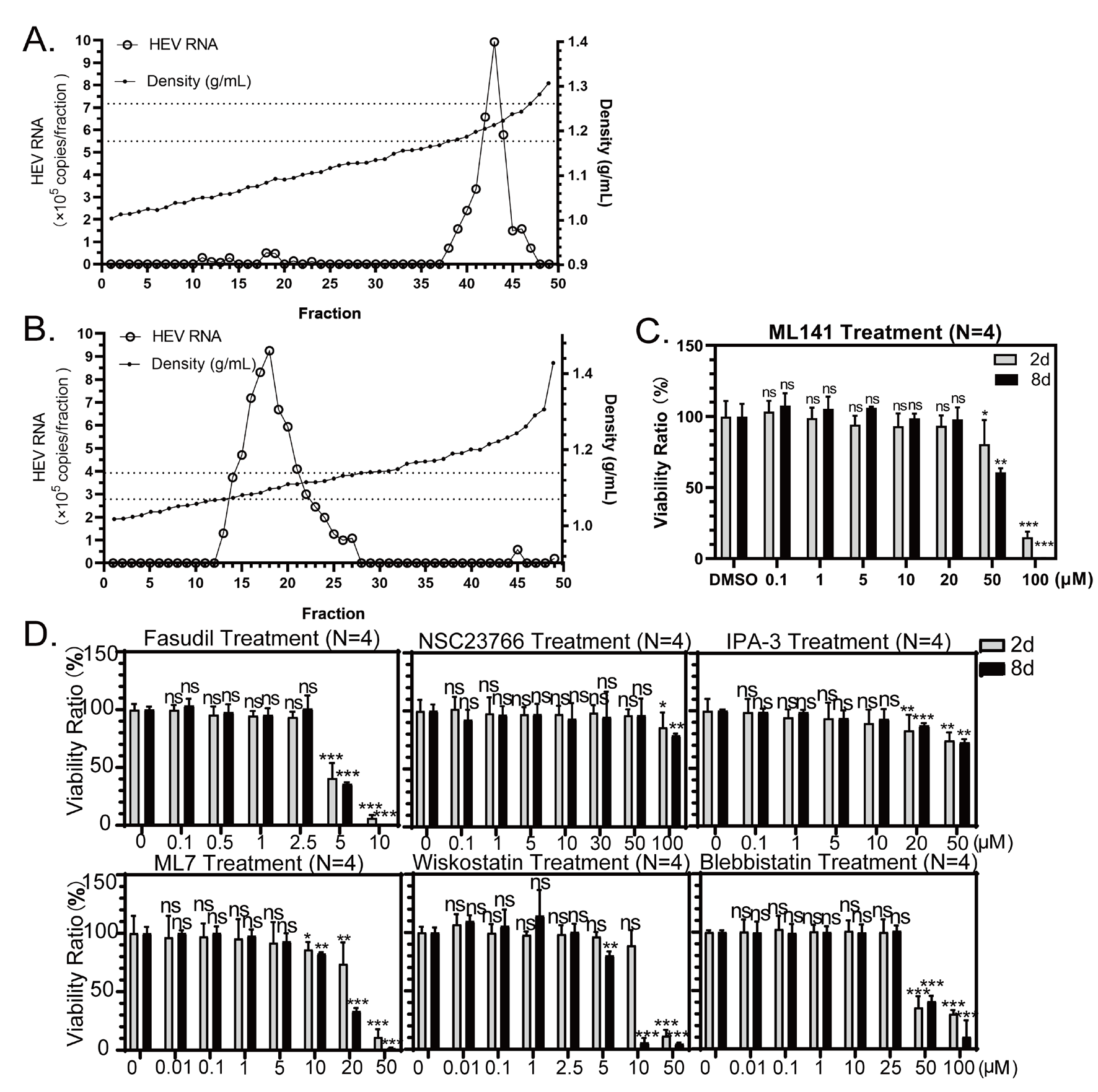

Supplement: Supplementary Figure 7 — Identification of HEV virions and cytotoxicity analysis of Rho family GTPases inhibitors in HepG2/C3A cells. Iodixanol gradient of non-enveloped HEV virions (A) and quasi-enveloped HEV virions (B). Iodixanol gradients of 10–50% w/v of HEV virions. HEV RNA infraction was determined by qPCR. Cytotoxicity analysis of Rho family GTPases inhibitors in HepG2/C3A cells (C,D). [file Image_7.TIF]
